# The “Hidden Hunger” Paradox Amidst a High-Energy Diet: A Cross-Sectional Assessment of an Adult Cohort Evaluated via a Professional Digital Dietary Tool in Russia

**DOI:** 10.3390/nu18132094

**Published:** 2026-06-26

**Authors:** Murat A. Kade, Inna Yu. Tarmaeva, Dmitry B. Nikityuk, Irina A. Lapik

**Affiliations:** 1 Federal Research Centre of Nutrition, Biotechnology and Food Safety, 109240 Moscow, Russia; tarmaeva@ion.ru (I.Y.T.); dimitrynik@mail.ru (D.B.N.); lapik_@inbox.ru (I.A.L.); 2 Nutrient Planner LLC, 192281 St. Petersburg, Russia; 3 Department of Operative Surgery and Topographic Anatomy, I.M. Sechenov First Moscow State Medical University (Sechenov University), 117198 Moscow, Russia

**Keywords:** hidden hunger, micronutrient intake shortfall, obesity paradox, digital nutrition, precision nutrition, technological losses, dietary patterns, dietary assessment

## Abstract

**Background/Objectives**: The obesity epidemic coexists with the phenomenon of “hidden hunger” (Type B malnutrition)—a micronutrient deficiency amidst a caloric excess. Traditional dietary assessment methods often distort the actual picture by ignoring technological losses during cooking, which necessitates the use of digital tools. **Methods**: A cross-sectional study (*N* = 3267) was conducted using the digital platform “NIAP”. The analysis was based on valid 3–7-day dietary records with algorithmic accounting for nutrient retention factors during thermal processing. The nutrient profiles of individuals with a normal body mass index (BMI) and obesity (BMI ≥ 30 kg/m^2^) were compared. **Results:** The epidemiology of intake shortfalls was highly prevalent and pronounced: 99.9% of the cohort had ≥1 inadequacy (with a mean negative deviation of −77.3% for vitamin D and −59.2% for Omega-3), and 61.5% exhibited ≥10 simultaneous multiple intake shortfalls. These inadequacy rates remained robust in a sensitivity analysis excluding under-reporters. The obesity group consumed significantly more energy, saturated fatty acids, added sugars, cholesterol, and sodium, but demonstrated a lower relative macronutrient intake (g/kg of body weight). Absolute fiber intake did not differ between the groups, indicating a decrease in its density per 1000 kcal in the diet of individuals with obesity; the intake of Omega-3 polyunsaturated fatty acids (PUFAs) showed a downward trend. The Na:K ratio was significantly higher in the obesity group (1.19 vs. 1.04, *p* < 0.001). Correlation analysis confirmed an inverse relationship between BMI and the overall nutrient density of the diet. **Conclusions**: A high-energy diet does not compensate for systemic micronutrient inadequacy among the evaluated cohort. Obesity is associated with a dietary imbalance favoring “empty calories” and pro-inflammatory components against a background of severe multiple dietary inadequacies. The integration of algorithmic dietary assessment that accounts for cooking losses is critical for objective diagnosis and personalized nutritional intervention.

## 1. Introduction

The global burden of malnutrition has undergone significant changes over recent decades. Classic protein-energy malnutrition has been superseded by an epidemic of obesity and metabolic disorders, which now affects most developed and developing countries [1,2]. However, against the background of excessive energy (macronutrient) intake, the medical community has encountered a growing problem of micronutrient inadequacy—a phenomenon termed “hidden hunger” (or Type B malnutrition) [3]. This paradox lies in the fact that achieving and even significantly exceeding individual caloric requirements does not guarantee the provision of essential vitamins, minerals, and amino acids to the body [4].

One of the main reasons for the development of “hidden hunger” is a global shift in dietary patterns characterized by a transition to the so-called “Western diet”. This type of diet is distinguished by high energy density due to added sugars, refined carbohydrates, and saturated fats (“empty calories”), while being critically deficient in dietary fiber and micronutrients [5,6]. As a result, a patient population emerges suffering simultaneously from obesity and severe multiple deficiency states, which synergistically increases the risks of cardiovascular diseases, type 2 diabetes mellitus, and other comorbid pathologies [7].

Despite the recognition of the problem, an accurate epidemiological assessment of the prevalence of “hidden hunger” in real-world clinical practice remains a challenging task. Traditional dietary assessment methods, such as food frequency questionnaires (FFQs) and 24 h dietary recalls (24 h recall), are prone to subjective bias and the phenomenon of energy under-reporting [8,9]. Moreover, the classic manual calculation of diets using food composition reference tables often ignores fundamental technological factors: changes in dish weight and the degradation of thermolabile nutrients during cooking. Ignoring these losses leads to a falsely optimistic assessment of nutritional status, masking actual deficiencies [10,11].

The solution to this problem is a shift toward precision nutrition methods using intelligent digital platforms (eHealth/mHealth). Modern automated algorithms allow not only the collection of Big Data on actual dietary intake at the population level but also the algorithmic accounting of technological nutrient losses, providing a detailed picture of nutrient adequacy [12,13]. Nevertheless, there is currently a lack of large-scale cross-sectional studies utilizing validated digital tools for a comprehensive assessment of the full spectrum of macro- and micronutrients (including fatty acid profile) in the adult population.

In this regard, the hypothesis of our study is that a high-energy diet associated with a high body mass index (BMI) has a critically low nutrient density, which fails to compensate for the inadequate intake of essential micronutrients and leads to the formation of widespread “hidden hunger”.

The aim of this study was to evaluate the macro- and micronutrient adequacy of the adult population’s diet using an automated digital platform, as well as to conduct a comparative analysis of dietary patterns and predictors of multiple deficiency states in individuals with a normal body weight and patients with obesity.

## 2. Materials and Methods

### 2.1. Study Design and Participants

This study was conducted using a retrospective cross-sectional design. Data were collected between February 2022 and October 2025 using the professional cloud-based digital platform “Scientific Dietary Analysis Tool” (NIAP, Nutrient Planner LLC, St. Petersburg, Russia). This tool provides an automated assessment of actual dietary intake by integrating food composition databases with nutrient recalculation algorithms.

The initial general population (screening base) consisted of 13,773 users who had completed a primary online questionnaire. To establish a reliable analytical database, the following inclusion criteria were applied: age 18 years and older, a fully completed anthropometric profile, and the maintenance of a valid electronic dietary record for a duration of 3 to 7 days. As a result, the analytical cohort comprised 3267 individuals (23.7% of the initial general population). The sample was characterized by a predominance of females (84.2%; *n* = 2751) over males (15.8%; *n* = 516). The mean age of the participants was 40.4 ± 11.4 years (median 39.0 years, interquartile range [IQR] 32.0–47.0).

It should be noted that the formed cohort is not representative of the entire adult population of the Russian Federation; rather, it reflects the demographic characteristics of users of digital dietary tracking platforms, featuring a characteristic predominance of women.

For each participant, a standardized set of parameters was extracted from the database:

- Demographics and anthropometry: sex, age, height, body weight, and circumferences (waist, hip, chest, and wrist).
- Calculated indices: body mass index (BMI), Pignet index (body type assessment), body adiposity index (BAI), and indices of abdominal obesity risk (waist-to-hip ratio [WHR] and waist-to-height ratio [WHtR]).
- Nutritional status and nutrient profile: average daily energy intake (in absolute values and kcal/kg of body weight), and the intake of macronutrients (including mono- and disaccharides, dietary fiber, fatty acids, and amino acids), essential vitamins, and minerals.
- Dietary patterns: average daily consumption (in grams) across 24 standardized food categories (including vegetables, fruits, bakery and confectionery products, fast food, meat, dairy products, etc.).
- Behavioral factors: sports activity level (categorical scale: “none”, “light sports”, “regular sports”, “active sports”), physical activity level (PAL, ranging from 1.4 to 2.2), and adhered dietary type (“unrestricted”, “vegetarian”, “gluten-free”, etc.).

The study was conducted in accordance with the principles of the Declaration of Helsinki [14]. Because the study utilized a retrospective analysis of fully anonymized and aggregated data, the requirement for additional approval by the local ethics committee was waived (Institutional Review Board waiver). Upon registering on the platform, all users accepted the user agreement and provided informed consent for the processing of their data for scientific purposes.

### 2.2. Digital Platform and Dietary Assessment

Anamnestic data collection and the assessment of actual dietary intake were carried out using the cloud-based platform “Scientific Dietary Analysis Tool” (NIAP) [15], developed with the participation of the Federal Research Centre of Nutrition, Biotechnology and Food Safety. The data collection process was organized through two channels. First, a specialist could generate a personalized link to an online questionnaire and dietary record for the patient. All data entered by the patient were transmitted in real time to the specialist’s personal account, ensuring continuous monitoring and timely feedback. Second, the data could be entered manually by the specialist directly during an in-person consultation.

To evaluate the nutrient profile of the diet, the built-in multi-component NIAP database was used, which contains over 7000 food items and 30,000 recipes. The chemical composition of the foods was derived from authoritative reference sources, including the United States Department of Agriculture (USDA) National Nutrient Database, the database of the Federal Research Centre of Nutrition and Biotechnology (Russia), as well as the national databases of Denmark and Israel [16,17]. The tool allows for the calculation of 75 macro- and micronutrients (including amino acid scores, fatty acid profiles, vitamins, minerals, and minor biologically active substances). As a clinical decision support system (CDSS), the NIAP platform functions as a reference-analytical tool. In accordance with national regulatory guidelines, its safety and algorithmic accuracy are verified not through prospective clinical trials (e.g., doubly labeled water or biomarker validation), but through strict adherence to the approved state guidelines for nutritional calculations (MR 2.3.1.0253-21) [18] and the integration of verified food composition databases.

A fundamental methodological feature of the NIAP algorithm is the automatic application of technological nutrient loss (retention) factors. Unlike traditional assessment methods (gross raw-weight methods), the system automatically calculates the change in the mass of ingredients during cold processing (peeling, chopping) and thermal culinary processing (yield factors for boiling, frying), as well as the specific degradation of thermolabile vitamins and minerals depending on the cooking method [19]. This allows for the reconstruction of the nutrient composition of the actually consumed prepared dishes.

### 2.3. Anthropometric and Nutritional Definitions

Body weight status was assessed based on the body mass index (BMI, kg/m^2^), calculated using the World Health Organization (WHO) classification [20]. Patients with a BMI ≥ 30.0 kg/m^2^ were classified into the obesity group. Additionally, the Pignet index, WHR (waist-to-hip ratio), and WHtR (waist-to-height ratio) were evaluated to assess abdominal risk.

The basal metabolic rate (BMR) was automatically calculated by the system as the average value from four validated equations that utilize different anthropometric approaches: the classic Harris–Benedict equation [21], the modern Mifflin-St Jeor equation [22], the World Health Organization (WHO) equation [23], and the body composition-based Katch–McArdle equation [24]. The resulting mean BMR value was then adjusted by the physical activity level (PAL) coefficient to estimate the individual daily energy requirement [23].

Nutritional adequacy was evaluated by comparing the actual daily intake of each nutrient with a personalized requirement. The baseline individual reference values (daily intake norms) in the NIAP platform were calculated based on the current “Norms of Physiological Requirements in Energy and Nutrients for Various Groups of the Population of the Russian Federation” (MR 2.3.1.0253-21) [18]. For nutrients not regulated in the national guidelines (specific trace elements, individual fatty acids, and vitamin-like substances), expanded international standards were applied: the Dietary Reference Intakes (DRIs) of the Institute of Medicine of the U.S. National Academies of Sciences [25].

A dietary micronutrient “inadequacy” (intake shortfall) was defined as a negative deviation of actual intake from the individual requirement by more than 10% (dev% < −10%). While a 10% threshold may appear conservative for short-term dietary records, it is a standard tolerance margin in clinical dietetics. More importantly, the actual mean deviations observed in our cohort were profoundly larger (e.g., −77.3% for vitamin D, −46.2% for folates), indicating that the high prevalence of inadequacy in this study is driven by severe systemic shortfalls rather than marginal threshold effects.

### 2.4. Statistical Analysis

Statistical processing and data visualization were performed using the Python programming language (version 3.13.7) and specialized data analysis libraries (pandas 2.3.3, numpy 2.3.3, scipy 1.16.2).

The normality of the distribution of continuous quantitative variables was tested using the Kolmogorov–Smirnov test (given the large sample size of *N* > 3000). Due to the large sample size (*N* = 3267) and the robustness of parametric tests to distribution deviations (according to the central limit theorem), continuous variables are described using the mean and standard deviation (M ± SD). For indicators with pronounced skewness, the median and interquartile range [IQR] are additionally provided. For key indicators, the bootstrap method was additionally applied to estimate the 95% confidence intervals (95% CIs) of medians and proportions.

Comparisons of continuous variables between two independent groups (e.g., individuals with a normal BMI and patients with obesity) were conducted using the non-parametric Mann–Whitney U test, as well as Welch’s *t*-test in cases of justified applicability. To evaluate qualitative nominal features (the frequency of detecting dietary inadequacies, the structure of the food basket), Pearson’s chi-square test (χ^2^) was applied.

Correlation analysis to identify relationships between dietary patterns, macronutrient intake, and anthropometric indices was performed by calculating Pearson correlation coefficients (r) and the non-parametric Spearman’s rank correlation coefficient (ρ). Differences and correlations were considered statistically significant at *p* < 0.05.

## 3. Results

### 3.1. Demographic and Anthropometric Profile

The final analytical cohort included 3267 individuals. The sample was characterized by a significant predominance of females (84.2%, *n* = 2751) over males (15.8%, *n* = 516). The mean age of the participants was 40.4 ± 11.4 years (median 39.0 years [IQR: 32.0–47.0]), predominantly encompassing young and middle-aged adults (18–59 years).

Analysis of the anthropometric data revealed a high prevalence of overweight and obesity in the studied population. The mean body mass index (BMI) was 25.7 ± 5.5 kg/m^2^ (median 24.5 [IQR: 21.4–28.7]). Normal body weight (BMI 18.5–24.9) was recorded in only half of the participants—49.9% (*n* = 1629). Overweight (BMI 25.0–29.9) was observed in 24.5% (*n* = 799) of the respondents. Obesity of varying severity (BMI ≥ 30.0) was identified in 20.3% (*n* = 664) of the patients. Severe underweight and underweight combined accounted for 5.4% (*n* = 174) of the sample. Further evaluation of abdominal risk indices showed that 52.7% of the men and 48.9% of the women had an elevated or high risk of metabolic disorders based on waist circumference. The baseline demographic and anthropometric characteristics of the studied cohort are presented in Table 1.

A baseline assessment of the cohort’s clinical profile revealed that 52.8% of participants had at least one chronic condition, including hypertension (5.2%), hypothyroidism (3.3%), and type 2 diabetes mellitus (2.2%). While metabolic disorders can influence dietary behavior, our data demonstrated that the phenomenon of multiple dietary inadequacies was consistently profound across all clinical subgroups. For instance, patients with type 2 diabetes and hypertension exhibited an average of 5.89 and 6.49 simultaneous vitamin intake shortfalls, respectively, which is closely comparable to the overall obesity group (6.12). This indicates that the nutrient-poor nature of the diet persists independently of the underlying metabolic diagnosis.

### 3.2. Macronutrient Profile and Energy Imbalance

Assessment of actual dietary intake demonstrated pronounced heterogeneity in energy consumption. The average daily energy intake was 1948 ± 585 kcal/day (median 1820 [IQR: 1521–2234]). When compared to individual daily requirements, it was found that only 32.1% (*n* = 1050) of the participants were in a state of energy balance (±10% of the norm). A caloric surplus (>+10% of the norm) was observed in 31.9% (*n* = 1043) of the respondents, while 35.9% (*n* = 1174) demonstrated an energy intake deficit.

Analysis of the macronutrient structure revealed a shift in macronutrient proportions toward an excessive intake of fats. The mean distribution of daily caloric intake was 20.0% from proteins, 42.2% from fats, and 37.8% from carbohydrates. Actual carbohydrate intake was below the recommended norm for 82.6% of the participants, whereas an excess fat intake (dev% > +10%) was recorded in 85.0% of the cohort.

When analyzing consumption patterns stratified by sex (Supplementary Table S1), males demonstrated significantly higher absolute energy intake compared to females (2417.1 ± 649.2 kcal/day vs. 1859.5 ± 527.0 kcal/day, *p* < 0.001), as well as higher absolute consumption of all major macronutrients. Relative energy intake was also slightly higher in males (28.9 ± 8.4 kcal/kg vs. 27.8 ± 8.7 kcal/kg, *p* < 0.001). Despite these differences in absolute volumes, the overarching trend of critical micronutrient inadequacy alongside an energy-dense dietary pattern remained consistent across both sexes.

### 3.3. Epidemiology of Micronutrient Inadequacies (“Hidden Hunger”)

To assess the true prevalence of “hidden hunger”, the dietary adequacy was analyzed using a targeted panel of clinically significant essential micronutrients. Trace elements highly dependent on regional water supply characteristics (fluorine, bromine, boron), as well as amino acids, were deliberately excluded from the analysis to prevent an artificial overestimation of nutritional inadequacy rates.

Analysis of individual dietary adequacy revealed a very high prevalence of multiple intake shortfalls within the studied cohort. Only 3 individuals out of 3267 (0.1%) had a fully balanced diet without micronutrient inadequacies. The absolute majority of the sample (99.9%, *n* = 3264) exhibited an inadequate intake of at least one essential nutrient.

Furthermore, 61.5% (*n* = 2009) of the studied population exhibited severe multiple dietary inadequacies—a simultaneous shortfall of 10 or more essential vitamins and minerals. The mean number of detected dietary inadequacies was 11.8 per patient (median 11 [IQR: 8–16]).

**Figure 1:**
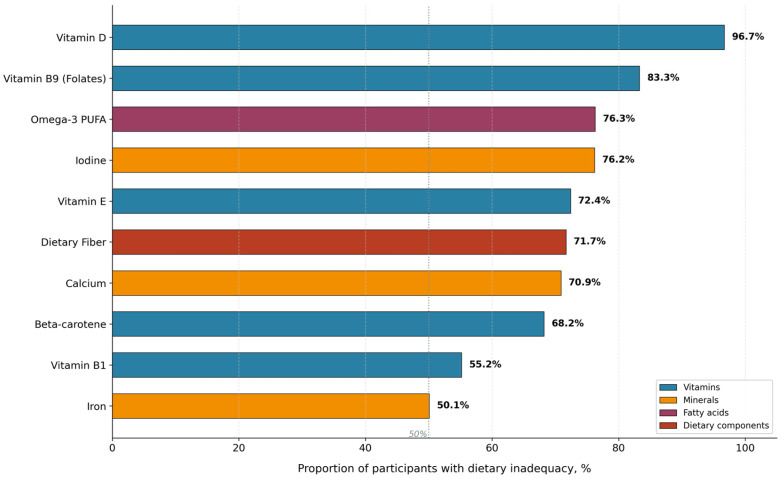
Prevalence of critical dietary micronutrient inadequacies in the study cohort (*n* = 3267). Notes: Intake shortfall was defined as deviation of actual intake from individual reference value by more than 10% (dev% < −10%). Abbreviations: PUFA—polyunsaturated fatty acids.

The most critical levels of nutritional inadequacy (detected in more than half of the cohort) were recorded for the following substances (Figure 1):

- Vitamins: The absolute negative leader was vitamin D (severe dietary inadequacy was recorded in 96.7% of the sample, which is expected given its limited natural food sources), followed by vitamin B9/folates (83.3%), vitamin E (72.4%), beta-carotene (68.2%), and vitamin B1 (55.2%).
- Minerals and trace elements: The most prevalent were dietary inadequacies in iodine (76.2%), calcium (70.9%), and iron (50.1%), while notable inadequacies were also observed for magnesium (39.7%, mean dev% = −27.4%) and chromium (14.7%, mean dev% = −34.4%).
- Other critically important nutrients: A dietary inadequacy in Omega-3 polyunsaturated fatty acids (PUFAs) was observed in 76.3% of the patients, and a shortage of dietary fiber was noted in 71.7%.

It is important to note that the detected dietary inadequacies were not marginal but rather pronounced. The mean deviation from the individual requirement (dev%) was −77.3% for vitamin D, −59.2% for Omega-3 PUFAs, −46.2% for folates (B9), −39.3% for dietary fiber, and −37.8% for calcium. This indicates that the diet of the studied population systematically lacks critically important nutrients in volumes that possess clinical significance.

### 3.4. Comparative Analysis of the Nutrient Profile in Normal BMI and Obesity

To evaluate the impact of body weight status on diet quality, a comparative analysis of the nutrient profile was conducted between the normal weight group (BMI 18.5–24.9 kg/m^2^; *n* = 1601) and the obesity group (BMI ≥ 30.0 kg/m^2^; *n* = 665) (Table 2).

It was found that patients with obesity consumed significantly more energy in absolute values (2131 ± 691 kcal/day vs. 1875 ± 533 kcal/day in the control group; *p* < 0.001). However, when recalculated per kilogram of body weight, the opposite effect was observed: relative energy intake in the obesity group was 27% lower (22.3 ± 6.8 kcal/kg vs. 30.6 ± 8.3 kcal/kg in the normal weight group; *p* < 0.001).

The identified pattern (higher absolute and lower relative intake) extended to all major macronutrients:

- Protein: 1.07 ± 0.44 g/kg in the obesity group vs. 1.48 ± 0.46 g/kg in the control group (*p* < 0.001).
- Fats: 1.03 ± 0.39 g/kg vs. 1.40 ± 0.48 g/kg (*p* < 0.001).
- Carbohydrates: 2.04 ± 0.78 g/kg vs. 2.85 ± 1.03 g/kg (*p* < 0.001).

This observation is explained by differences in the metabolic activity of tissues: adipose tissue consumes significantly less energy per unit of mass compared to muscle tissue, which mathematically leads to a decrease in the average caloric requirement per kilogram of total body weight as the proportion of fat mass increases.

Although an increase in the absolute intake of certain nutrients is mathematically expected alongside a higher overall energy intake, the clinical significance of the observed paradox lies in the severe disproportion of relative intake and nutrient density. Despite the lower relative intake of nutrients, the absolute intake of “empty calories” and pro-inflammatory components was significantly higher in the obesity group. Specifically, they consumed significantly more saturated fatty acids (SFAs) (32.8 ± 14.5 g/day vs. 28.3 ± 11.6 g/day in the control group, *p* < 0.001), added sugars (73.2 ± 37.4 g/day vs. 66.5 ± 31.6 g/day, *p* < 0.001), cholesterol (442.6 ± 213.6 mg/day vs. 393.8 ± 184.9 mg/day, *p* < 0.001), and sodium (3514.3 ± 2057.1 mg/day vs. 2831.8 ± 1333.5 mg/day, *p* < 0.001).

Furthermore, the caloric surplus did not lead to an improved supply of protective nutrients. Dietary fiber intake did not differ statistically between the groups (20.1 ± 10.3 g/day in obesity vs. 20.7 ± 9.6 g/day in normal weight, *p* = 0.082), indicating a lower fiber density per 1000 kcal in the diet of individuals with obesity. The intake of anti-inflammatory Omega-3 fatty acids showed a downward trend in individuals with obesity (1.37 ± 1.24 g/day vs. 1.56 ± 1.67 g/day, *p* = 0.054). A pronounced electrolyte imbalance was also recorded in the obesity group—the sodium-to-potassium (Na:K) ratio was 1.19 ± 0.56 compared to 1.04 ± 0.51 in the normal BMI group (*p* < 0.001).

These trends are supported by the results of the correlation analysis. A significant inverse relationship was established between BMI and relative protein intake (ρ = −0.440, *p* < 0.001), as well as carbohydrate intake (ρ = −0.414, *p* < 0.001) in g/kg of body weight. At the same time, the Pignet index (a marker of an asthenic body type) had a pronounced positive correlation with the relative caloric content of the diet (ρ = 0.504, *p* < 0.001), which indicates a higher energy density of the diet per kilogram of body weight in individuals with an asthenic morphotype compared to individuals with obesity.

### 3.5. Dietary Patterns and Sources of “Empty Calories”

**Figure 2:**
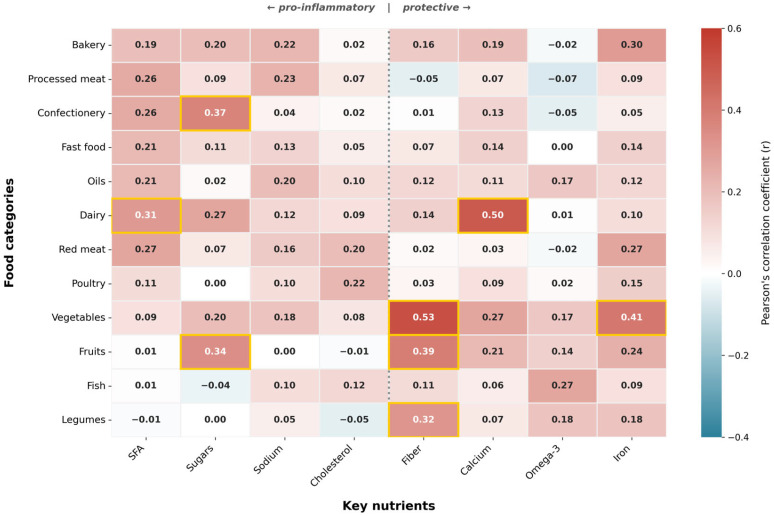
Correlation heatmap between major food categories and key pro-inflammatory and protective nutrients. Notes: Pearson’s correlation coefficients (r) are shown. Color scale: red (positive correlation) through white (r ≈ 0) to blue (negative correlation). Strong associations (|r| > 0.3) are highlighted with gold borders. The dotted line separates pro-inflammatory (SFA, sugars, sodium, cholesterol) and protective (fiber, potassium, Omega-3, iron) nutrients. Abbreviations: SFA—saturated fatty acids.

To identify the dietary mechanisms underlying the observed nutrient imbalance, a correlation analysis was conducted between the consumption of 24 food categories and key nutrients across the entire studied cohort (*N* = 3267) (Figure 2).

The analysis revealed clear and stable dietary patterns explaining the simultaneous development of obesity and micronutrient inadequacy.

To specifically elucidate the drivers of nutrient imbalance within the high-risk group, an additional correlation analysis was performed exclusively for the obesity sub-cohort (BMI ≥ 30 kg/m^2^; Supplementary Figure S1). The pro-inflammatory dietary patterns observed in the overall cohort were fully replicated within this subgroup, confirming that energy-dense, ultra-processed food categories act as primary drivers of micronutrient dilution regardless of the baseline BMI.

Regarding dietary preferences, the majority of the cohort (74.8%) followed an unrestricted diet, while others adhered to restrictive patterns, primarily lactose-free (7.1%), gluten-free (5.3%), vegetarian (1.5%), and vegan (0.6%). Interestingly, the specific type of diet did not significantly alter the overall burden of “hidden hunger”. The average number of micronutrient intake shortfalls remained critically high across all groups (e.g., 6.4 vitamin inadequacies per person in the unrestricted group, compared to 5.8 in the vegan group). This indicates that popular restrictive diets in this population are generally unbalanced and do not eliminate the problem of low nutrient density.

Sources of Energy Density and Pro-inflammatory Components:

The analysis showed that the increase in caloric intake and the consumption of nutrients associated with metabolic risks are closely linked to a narrow group of foods.

- Saturated fatty acids (SFAs): The strongest predictors of high SFA intake were dairy products (r = 0.310), meat (r = 0.273), and sausages/processed meats (r = 0.260). This pattern, combining animal fats and industrially processed foods, is a classic marker of the “Western diet”.
- Sugars: Interestingly, the two main sources of sugars in the diet had almost identical correlation strengths: confectionery (r = 0.373) and fruits (r = 0.345). However, their nutrient “package” differs radically: the consumption of confectionery is also strongly correlated with SFAs, whereas fruit consumption is one of the leading predictors of dietary fiber intake (r = 0.389).
- Cholesterol: The primary and overwhelming dietary determinant of cholesterol intake in the studied cohort was eggs, which showed an extremely strong correlation (r = 0.760). Secondary contributions came from offal (r = 0.241) and poultry (r = 0.219).
- Sodium: The main sources of “hidden” sodium in the diet were sausages/processed meats (r = 0.227) and bakery products (r = 0.218).

It is important to note that the identified correlations are supported by the actual consumption structure. For instance, bakery products are present in the diet of 90.8% of the participants (mean intake among consumers is 53.7 g/day), confectionery in 66.2% (32.6 g/day), and sausages/processed meats in 40.4% (34.0 g/day). Although sausages have a lower proportion of consumers, their high energy density and content of hidden fats/sodium make them a significant driver of the pro-inflammatory dietary pattern. At the same time, the consumption of these categories correlates weakly with the intake of dietary fiber and vitamins, which confirms their role as sources of “empty calories” at the population level.

Sources of Protective Nutrients:

In contrast, the consumption of key protective nutrients was associated with entirely different food categories.

- Dietary fiber: The main contributors to dietary fiber intake were vegetables (r = 0.528), fruits (r = 0.389), and leafy greens (r = 0.383).
- Calcium: Although the dominant source of calcium was dairy products (r = 0.498), significant contributions also came from plant-based sources, such as seeds (r = 0.285) and vegetables (r = 0.274).
- Iron: An important feature of the studied cohort is that the strongest statistical predictors of overall iron intake were plant-based sources: vegetables (r = 0.406), seeds (r = 0.389), and leafy greens (r = 0.330). The correlation with meat consumption (r = 0.265) was noticeably weaker.
- Omega-3: The primary source of anti-inflammatory Omega-3 fatty acids was fish (r = 0.271). However, a noticeable contribution was also made by a cluster of plant-based foods, including nuts (r = 0.201), legumes (r = 0.184), and oils (r = 0.173).

An opposite picture is observed for foods that form the nutrient-dense part of the diet. Vegetables, fruits, and leafy greens are present in the daily menus of almost the entire sample (99.8%, 91.8%, and 93.1%, respectively). However, their actual consumption volumes (an average of 215.0 g of vegetables and 110.4 g of fruits per day) remain below clinical recommendations, which fails to compensate for the high proportion of refined carbohydrates and animal fats in the diet. This quantitative deficit of plant-based components serves as a direct dietary explanation for the identified widespread prevalence of inadequacies in dietary fiber (71.7%), vitamin B9/folates (83.3%), and β-carotene (68.2%). Thus, even with a high formal penetration of protective foods into the diet, their insufficient actual volume does not provide the required nutrient density, entrenching the phenomenon of “hidden hunger” at the population level.

Therefore, a structural analysis of the food basket reveals a fundamental dichotomy in the diet of the studied population: a high energy density is formed by mass-consumed but nutrient-poor categories (bakery, confectionery, and sausages/processed meats), whereas the intake of essential micronutrients critically depends on the volume of vegetables, leafy greens, and fruits, the actual consumption of which is insufficient to correct the identified multiple dietary inadequacies. This disproportion is the key dietary mechanism explaining the “hidden hunger” paradox: excessive caloric intake is not accompanied by an adequate supply of vitamins and minerals, which creates a metabolic basis for the progression of obesity and comorbid conditions.

## 4. Discussion

### 4.1. Interpretation of Main Findings

The obtained data robustly confirm the proposed hypothesis: a high-energy diet associated with an elevated BMI does not compensate for the inadequate intake of essential micronutrients but rather exacerbates it due to a critically low nutrient density. The identified exceptionally high prevalence of dietary inadequacies demonstrates that the phenomenon of “hidden hunger” is no longer a marginal issue and may reflect a broader trend in modern dietary pattern. A critically important aspect is not only the high frequency but also the pronounced severity of the identified deviations. Rather than merely bordering on suboptimal intake, the profound shortfalls observed for critical nutrients—such as vitamin D, Omega-3 PUFAs, folates, and dietary fiber—shift the dietary pattern from the category of “suboptimal intake” to the zone of clinically significant deficiency, capable of systemically disrupting metabolic homeostasis, sustaining chronic low-grade inflammation, and reducing the body’s adaptive reserve [4,7,26].

A comparative analysis of the normal BMI and obesity groups quantitatively reveals the mechanism of this phenomenon. A fundamental discrepancy was identified: despite a statistically significantly higher absolute intake of energy and macronutrients, their relative intake (in g/kg of body weight) in the obesity group is critically reduced. The caloric surplus is formed primarily by pro-inflammatory components (added sugars, sodium, SFAs, cholesterol), whereas protective nutrients (dietary fiber, Omega-3) remain at the level of the control group or lower. This structural disproportion confirms that, in the diet of individuals with obesity, energy-dense, ultra-processed foods functionally displace micronutrient-rich food matrices, creating a direct dietary mechanism for the coexistence of excess body weight and severe nutritional inadequacy within a single population.

### 4.2. Comparison with Global Data

The scale and profile of the identified dietary inadequacies are consistent with global epidemiological trends; however, they demonstrate more pronounced values compared to data from national dietary surveys. Analysis of large representative samples, including NHANES data (USA) and the generalized results of national dietary surveys in European countries, records a high prevalence of inadequate intake of vitamin D, folates, calcium, dietary fiber, and other nutrients, reaching 40–90% depending on the specific nutrient, applied reference values (EAR/AI), and demographic stratification [27,28]. In our sample, the observed intake shortfall rates for these critical nutrients significantly exceed these population estimates. This dissociation is explained by methodological rigor: the algorithmic accounting of technological losses during cooking and the use of an expanded panel of 75 nutrients provide a physiologically more accurate reconstruction of actual intake than standard questionnaire methods, which often overestimate real consumption. Furthermore, the specific nature of the studied diet reflects a global shift toward energy-dense but nutrient-poor patterns, which, in the context of the Russian Federation, occurs in the absence of a systemic mandatory food fortification program and a low prevalence of dietary supplement use, additionally amplifying the risk of multiple micronutrient inadequacies and subsequent deficiency states [29].

### 4.3. Dietary Risk Factors for Comorbidity

The nutrient profile associated with obesity in this cohort represents a clearly defined dietary matrix for the risk of developing cardiometabolic disorders. The statistically significant increase in sodium intake combined with a shift in the Na:K ratio in the obesity group directly correlates with the risk of endothelial dysfunction and arterial hypertension [30]. Concurrently, inadequate dietary fiber intake (which is substantially below the WHO recommendations) and a pronounced intake shortfall in Omega-3 PUFAs form a pro-inflammatory and insulin-resistant metabolic environment [26]. In our cohort, notable dietary shortfalls were identified for magnesium and chromium. From a pathophysiological perspective, these specific inadequacies could potentially contribute to the reinforcement of the “obesity–hidden hunger” cycle. Magnesium is a vital cofactor for glucose transport and insulin secretion; its chronic deficiency has been associated in the literature with impaired cellular insulin sensitivity and low-grade systemic inflammation. Similarly, chromium is essential for optimal insulin receptor activity and glucose tolerance, functioning as a cofactor for chromodulin. Its depletion is hypothesized to impair insulin action, potentially contributing to compensatory sugar cravings and dysregulated satiety signals. Together, these micronutrient shortfalls may help sustain a feedback loop where dietary inadequacy could exacerbate insulin resistance, thereby promoting compensatory overeating and subsequent weight gain. Correlation analysis details this mechanism: sodium and SFA loads are closely associated with the consumption of sausages/processed meats and bakery products, whereas the intake of dietary fiber and essential micronutrients almost entirely depends on the volume of vegetables, leafy greens, and fruits, the actual consumption of which in the sample is insufficient to compensate for the energy imbalance. This dietary dichotomy explains why weight gain in the studied population is caused not simply by a caloric excess but may be closely linked by a nutrient-poor, pro-inflammatory food environment that disrupts metabolic homeostasis, microbiota composition, and satiety signals, potentially reinforcing the “obesity-hidden hunger” cycle.

### 4.4. Practical Significance and the Role of Digitalization

From methodological and clinical perspectives, the present study highlights the critical limitations of traditional dietary assessment tools and the need to transition to digital nutrition frameworks. Standard food frequency questionnaires (FFQs) and manual calorie-tracking apps systematically overestimate nutrient adequacy by ignoring thermal degradation and weight losses during processing, which, when calculating recipes, can lead to an overestimation of the content of thermolabile nutrients [10,31]. The algorithmic integration of nutrient retention factors in the NIAP platform provides a physiologically detailed reconstruction of actual micronutrient delivery, which likely explains the contrast between our results and the historically optimistic reports from user-generated dietary records. In clinical practice, these findings support a paradigm shift: routine patient assessment should transition from calculating BMI and total caloric intake to the comprehensive profiling of micronutrient status. Digital eHealth/mHealth tools capable of automated, granular dietary logging and real-time mapping of dietary inadequacies should ideally be integrated into preventive medicine pathways to ensure early, targeted nutritional interventions before metabolic decompensation occurs. In terms of clinical application, the NIAP platform could be integrated beyond the research context into routine preventive health examinations (check-ups). Rather than relying solely on BMI and generalized dietary advice, clinicians could potentially utilize such digital dietary analysis tools for rapid, automated 3–5-day dietary profiling prior to patient consultations. This approach could allow for the immediate identification of specific nutrient gaps—including critical vitamin D, folate, fiber, and mineral shortfalls—and could enable primary care physicians and nutritionists to deliver targeted, personalized dietary prescriptions. Such early digital interventions may potentially help disrupt the metabolic cycle of obesity before irreversible comorbid complications develop.

### 4.5. Study Limitations

Several methodological limitations must be considered when interpreting the results. First, the cohort demonstrates a pronounced gender imbalance (84.2% women). Furthermore, the study population represents a self-selected group of digital platform users. This inherent selection bias is consistent with the established demographics of eHealth platform and digital wellness service users, but significantly limits the generalizability of the findings to the broader or male population.

Second, formal calculation revealed classic signs of energy under-reporting in 41.0% of the participants based on the standard epidemiological threshold (Energy < 0.8 × BMR × PAL). However, in the context of using a professional mHealth platform, this indicator cannot be interpreted solely as an accounting error. Given that 44.8% of the studied cohort had overweight or obesity, and data collection occurred within the framework of nutritional counseling, a significant portion of this group was most likely on a deliberate hypocaloric diet for weight loss. Unlike traditional retrospective questionnaires, precise portion weighing for a digital dietary record significantly reduces the frequency of accidentally omitted dishes. Nonetheless, self-reported dietary assessment, even with digital support and weighing protocols, remains inherently susceptible to cognitive and reporting biases, such as recall errors or socially desirable reporting. To rigorously test the validity of our findings against potential reporting bias, we performed a sensitivity analysis excluding all identified under-reporters (*n* = 1339, 41.0%; remaining sub-cohort *n* = 1928). In this validated, energy-sufficient sub-cohort (mean energy intake 2216 ± 576 kcal/day), the prevalence of critical dietary inadequacies remained virtually unchanged and profoundly high: vitamin D inadequacy was 95.9% (vs. 96.7% in the full cohort), vitamin B9/folates was 78.2% (vs. 83.3%), Omega-3 PUFAs was 72.1% (vs. 76.3%), calcium was 60.1% (vs. 70.9%), and dietary fiber was 61.8% (vs. 71.7%). The average number of simultaneous micronutrient intake shortfalls per patient remained at 9.8. These results demonstrate that the observed “hidden hunger” is a robust, systemic quality issue of the food basket itself, rather than an artifact of energy under-reporting. Consequently, even in the presence of some under-reporting of absolute food volumes, key markers of diet quality, such as nutrient density and relative ratios (Na:K, SFAs to dietary fiber), remain methodologically robust indicators that reliably reflect the fundamental dietary trends in the studied population.

Additionally, this study assessed exclusively dietary (alimentary) intake from food sources and did not control for the use of dietary supplements or medications (e.g., metformin, GLP-1 receptor agonists, thyroid hormones). Importantly, we must explicitly acknowledge the lack of biomarker validation in our methodology. Because the study relies solely on self-reported dietary intake data, it must be clearly stated that calculated dietary inadequacies do not necessarily reflect the actual physiological or biochemical nutritional status of the individuals. However, this aligns with the primary objective of our study: to evaluate the baseline nutritional quality of the diet itself, rather than the final biochemical status of the patients. The potential use of supplements does not negate the fundamental finding that the underlying dietary pattern is critically nutrient-poor. Moreover, while the demographic composition (predominantly women around 40 years of age) may introduce unmeasured metabolic confounders such as menopausal transition, the NIAP platform dynamically adjusts individual baseline requirements (BMR and micronutrient norms) based on real-time age, sex, and anthropometric data, which partially mitigates the impact of these physiological shifts on the calculated adequacy of the diet.

Third, the cross-sectional design of the study precludes the establishment of causal relationships. Consequently, we cannot definitively determine whether poor diet quality preceded and contributed to the development of obesity, or whether obesity itself influenced dietary reporting. Furthermore, individuals with obesity may have already modified their dietary patterns due to previous nutritional counseling or active weight-loss attempts, which could confound the observed relationships. While we observed robust associations, longitudinal cohort studies are required to verify these temporal dependencies and clarify the direction of causality.

Finally, several trace elements highly dependent on regional water composition (fluorine, bromine, boron), as well as the specific amino acid score, were deliberately excluded from the primary inadequacy analysis to prevent confounding and will be addressed in a separate methodological publication. Despite these limitations, the large sample size, standardized digital assessment, and rigorous statistical validation form a solid evidence base for understanding the modern “diet-obesity” paradox.

## 5. Conclusions

The results of the present study robustly confirm the hypothesis of a pronounced “hidden hunger” paradox in the context of a modern high-energy diet among users of a digital dietary platform. Achieving an energy surplus and the development of obesity do not compensate for the critical inadequacy of essential micronutrients, as the excessive caloric intake is formed primarily by “empty calories” (sugars, saturated fatty acids, sodium) alongside a systemic intake shortfall of dietary fiber, vitamins, and minerals. The identified widespread prevalence of multiple dietary inadequacies (99.9% of the cohort) and their clinically significant severity indicate the need for a fundamental revision of approaches to the prevention of diet-related diseases. The integration of precision nutrition digital platforms, providing automated accounting of technological losses and granular monitoring of nutrient density, should become the standard of preventive medicine to ensure the timely, personalized correction of nutritional status and to break the “obesity–nutritional inadequacy” cycle.

## Figures and Tables

**Table 1:** Baseline demographic and anthropometric characteristics of the study cohort (*n* = 3267).

Parameter	Value
Sex, *n* (%)	
Female	2751 (84.2)
Male	516 (15.8)
Age, years	40.4 ± 11.4; 39.0 [32.0–47.0]
Height, cm	167.1 ± 8.0
Body weight, kg	71.8 ± 17.6
Body mass index, kg/m^2^	25.7 ± 5.5; 24.5 [21.4–28.7]
BMI categories, *n* (%)	
Underweight (<18.5)	174 (5.4)
Normal weight (18.5–24.9)	1629 (49.9)
Overweight (25.0–29.9)	799 (24.5)
Obesity class I (30.0–34.9)	436 (13.3)
Obesity class II (35.0–39.9)	141 (4.3)
Obesity class III (≥40.0)	88 (2.7)
Waist circumference, cm	83.8 ± 15.3 ^1^
Abdominal risk by waist circumference, *n* (%) ^2^	
Men: Increased risk (≥94 cm)	82 (19.9)
Men: High risk (≥102 cm)	135 (32.8)
Women: Increased risk (≥80 cm)	477 (19.2)
Women: High risk (≥88 cm)	737 (29.7)

^1^ data presented for subsample with measured waist circumference (*n* = 2893); ^2^ data shown for men (*n* = 412) and women (*n* = 2481) with available measurements. Abbreviations: BMI—body mass index; values are presented as mean ± standard deviation; median [interquartile range: Q1–Q3]; *n* (%)—absolute number (percentage of sample).

**Table 2:** Comparison of dietary intake between participants with normal BMI (18.5–24.9 kg/m^2^) and obesity (≥30 kg/m^2^).

Parameter	Normal BMI (18.5–24.9 kg/m^2^), *n* = 1601	Obesity (≥30 kg/m^2^), *n* = 665	*p*-Value
Energy intake, kcal/day	1874.7 ± 533.5	2131.3 ± 691.4	<0.001 ***
Energy intake, kcal/kg/day	30.6 ± 8.3	22.3 ± 6.8	<0.001 ***
Protein, g/day	90.7 ± 29.9	102.0 ± 39.3	<0.001 ***
Protein, g/kg/day	1.48 ± 0.46	1.07 ± 0.44	<0.001 ***
Total fat, g/day	85.8 ± 29.5	98.6 ± 38.6	<0.001 ***
Total fat, g/kg/day	1.40 ± 0.48	1.03 ± 0.39	<0.001 ***
Saturated fatty acids, g/day	28.3 ± 11.6	32.8 ± 14.5	<0.001 ***
Saturated fatty acids, g/kg/day	0.46 ± 0.19	0.34 ± 0.15	<0.001 ***
Carbohydrates, g/day	174.3 ± 65.0	194.4 ± 77.9	<0.001 ***
Carbohydrates, g/kg/day	2.85 ± 1.03	2.04 ± 0.78	<0.001 ***
Sugars, g/day	66.5 ± 31.6	73.2 ± 37.4	0.030 *
Dietary fiber, g/day	20.7 ± 9.6	20.1 ± 10.3	0.082
Cholesterol, mg/day	393.8 ± 184.9	442.6 ± 213.6	0.016 *
Sodium, mg/day	2831.8 ± 1333.5	3514.3 ± 2057.1	<0.001 ***
Potassium, mg/day	2878.1 ± 1032.8	3077.5 ± 1420.2	<0.001 ***
Na:K ratio	1.04 ± 0.51	1.19 ± 0.56	<0.001 ***
Omega-3 PUFA, g/day	1.56 ± 1.67	1.37 ± 1.24	0.054
Iron, mg/day	15.3 ± 6.6	16.6 ± 6.3	<0.001 ***

Notes: data are presented as mean ± standard deviation; group comparisons were performed using Welch’s *t*-test or Mann–Whitney U-test depending on distribution normality; * *p* < 0.05; *** *p* < 0.001. Abbreviations: PUFA—polyunsaturated fatty acids; Na:K—sodium-to-potassium ratio.

## Data Availability

The anonymized data presented in this study are available on reasonable request from the corresponding author due to privacy and ethical restrictions regarding personal health information.

## Supplementary Materials

The following supporting information can be downloaded at: https://www.mdpi.com/article/10.3390/nu18132094/s1, Figure S1: Correlation heatmap between major food categories and key pro-inflammatory and protective nutrients specifically for the obesity sub-cohort (BMI ≥ 30 kg/m^2^); Table S1: Comparison of dietary intake between male and female participants.

## Abbreviations

The following abbreviations are used in this manuscript:

BMI	Body Mass Index
NIAP	Scientific Dietary Analysis Tool
PUFA	Polyunsaturated Fatty Acids
SFA	Saturated Fatty Acids
WHR	Waist-to-Hip Ratio
WHtR	Waist-to-Height Ratio
BAI	Body Adiposity Index
BMR	Basal Metabolic Rate
PAL	Physical Activity Level
DRI	Dietary Reference Intakes
EAR	Estimated Average Requirement
AI	Adequate Intake
FFQ	Food Frequency Questionnaire
IQR	Interquartile Range
SD	Standard Deviation
Na:K	Sodium-to-Potassium Ratio
WHO	World Health Organization
USDA	United States Department of Agriculture
