# Supplementary material for: The “Hidden Hunger” Paradox Amidst a High-Energy Diet: A Cross-Sectional Assessment of an Adult Cohort Evaluated via a Professional Digital Dietary Tool in Russia
Source: Nutrients. 2026 Jun 26;18(13):2094. doi: 10.3390/nu18132094 (PMC13363122; doi:10.3390/nu18132094)
Supplement: Supplementary file 1 [file nutrients-18-02094-s001.zip › nutrients-4336754-supplementary table S1.pdf]

Supplementary Table S1. Comparison of dietary intake between male and female participants.

Parameter	Males (n = 516)	Females (n = 2751)	p-value
Energy intake, kcal/day	2417.1 ± 649.2	1859.5 ± 527.0	<0.001***
Energy intake, kcal/kg/day	28.9 ± 8.4	27.8 ± 8.7	<0.001***
Protein, g/day	119.6 ± 40.9	89.0 ± 28.6	<0.001***
Protein, g/kg/day	1.42 ± 0.49	1.34 ± 0.48	<0.001***
Total fat, g/day	106.4 ± 38.3	86.1 ± 30.1	<0.001***
Total fat, g/kg/day	1.27 ± 0.46	1.29 ± 0.48	0.701
Saturated fatty acids, g/day	35.1 ± 14.4	28.4 ± 11.5	<0.001***
Saturated fatty acids, g/kg/day	0.42 ± 0.17	0.43 ± 0.18	0.910
Carbohydrates, g/day	228.7 ± 75.9	171.1 ± 63.9	<0.001***
Carbohydrates, g/kg/day	2.77 ± 1.07	2.57 ± 1.03	<0.001***
Sugars, g/day	76.0 ± 37.1	66.6 ± 32.8	<0.001***
Dietary fiber, g/day	23.3 ± 10.4	19.8 ± 9.4	<0.001***
Cholesterol, mg/day	484.4 ± 248.7	395.7 ± 182.7	<0.001***
Sodium, mg/day	3,802.8 ± 1919.8	2,884.0 ± 1601.4	<0.001***
Potassium, mg/day	3,371.6 ± 1119.4	2,859.6 ± 1132.5	<0.001***
Na:K ratio	1.18 ± 0.63	1.06 ± 0.56	<0.001***
Omega-3 PUFA, g/day	1.56 ± 1.56	1.50 ± 1.54	0.203
Iron, mg/day	19.5 ± 7.6	14.9 ± 5.7	<0.001***

Notes: data are presented as mean ± standard deviation; *** p<0.001.
