# Supplementary figures and images for: The “Hidden Hunger” Paradox Amidst a High-Energy Diet: A Cross-Sectional Assessment of an Adult Cohort Evaluated via a Professional Digital Dietary Tool in Russia
Source: Nutrients. 2026 Jun 26;18(13):2094. doi: 10.3390/nu18132094 (PMC13363122; doi:10.3390/nu18132094)

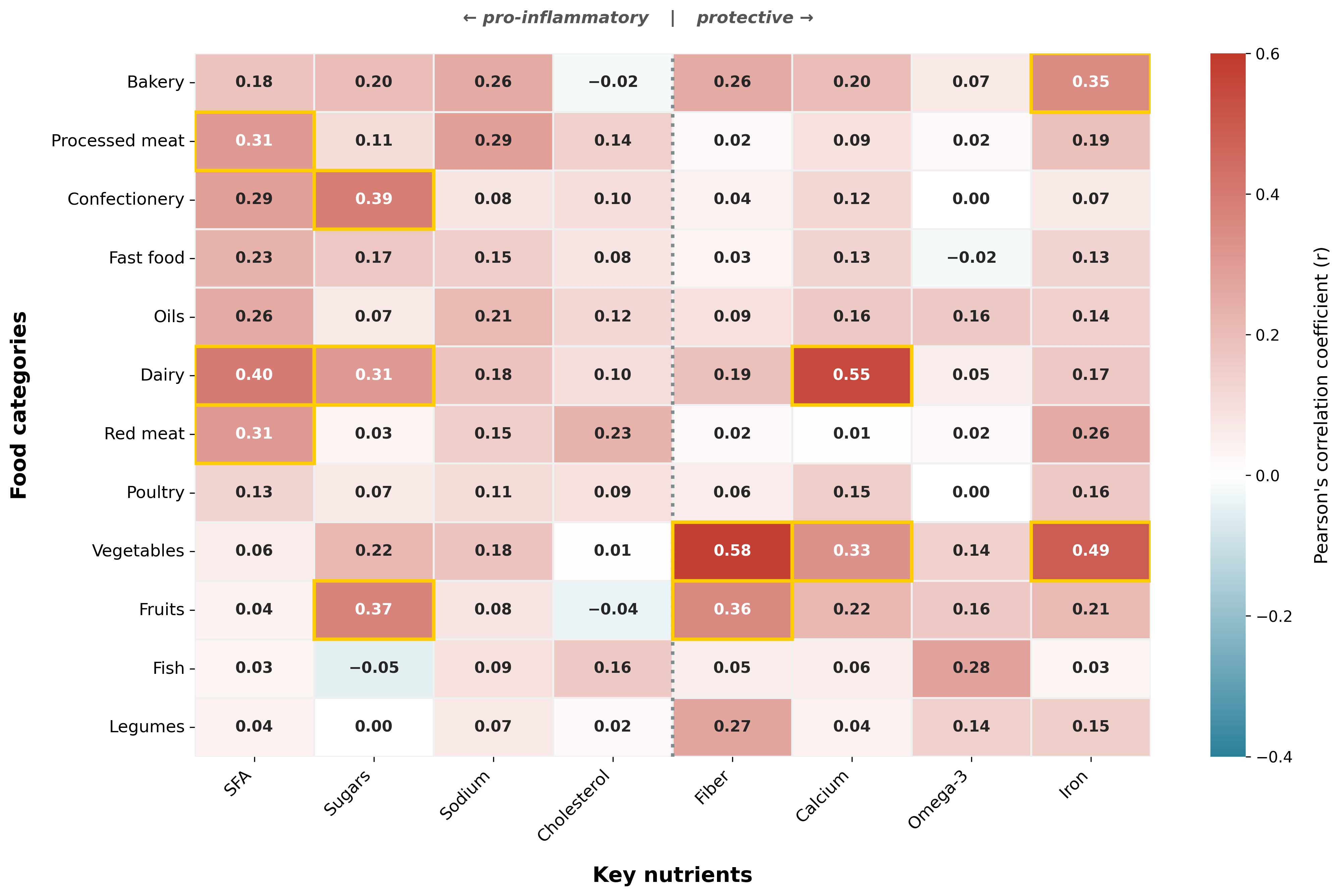

Supplement: Supplementary file 1 [file nutrients-18-02094-s001.zip › nutrients-4336754-supplementary.png]
